# An Innovative Model of Bronchopulmonary Dysplasia in Premature Infants

**DOI:** 10.3389/fped.2020.00271

**Published:** 2020-05-27

**Authors:** Xiaoyue Zhang, Xiaoyun Chu, Bowen Weng, Xiaohui Gong, Cheng Cai

**Affiliations:** Department of Neonatology, Shanghai Children's Hospital, Shanghai Jiao Tong University, Shanghai, China

**Keywords:** premature, rats, hyperoxia, animal model, bronchopulmonary dysplasia

## Abstract

Bronchopulmonary dysplasia (BPD) is one of the common chronic lung diseases (CLD) of premature infants, which causes unpredictable consequences to the family and society. Therefore, the pathogenesis and prevention methods of BPD are the focus of current research, and the establishment of an effective and appropriate animal model of BPD in premature infants is the key to the research. In this study, premature rats were exposed to hyperoxia environment. Compared with the air group, the body weight and alveolar radiation count of the hyperoxia group decreased significantly, but there was no significant difference in body length. HE staining was used to observe the pathological changes of BPD in the lung tissue. The above results proved that under the hyperoxia condition, the BPD animal model of premature infants was successfully established, which provided a new choice for the future research of BPD.

## Introduction

In recent years, the birth rate of premature infants, especially very low birth weight infants (VLBWI) and extremely low birth weight infants (ELBWI), has increased year by year, which is estimated to account for 11% of all births (1). Bronchopulmonary dysplasia (BPD) is one of the serious diseases that bring long-term adverse prognosis to premature infants. At present, the treatment of BPD is mainly in accordance with the symptoms. Therefore, it is very important to study the pathogenesis, effective prevention, and treatment methods of BPD. The classic pathological features of BPD are severe airway epithelial lesions, extensive metaplasia, and hyperplasia of airway mucous epithelium, extensive alveolar septal fibrosis, and pulmonary vascular remodeling (2), while the *new BPD* emphasizes the simplification of alveolar structure, pulmonary vascular malformation, and interstitial cells and / or fibrous hyperplasia (3). In the study of BPD, the establishment of animal models is essential. The more commonly used animal models are mice, rats, rabbits, sheep, and baboons (4–8). At present, most people think that oxidative stress of lung tissue caused by hyperoxia exposure is an important reason for the occurrence and development of BPD (9), and preterm is an important risk factor for the occurrence of BPD (10). Thanks to BPD brings many harmful effects to family and society, it is the main work to seek effective prevention and treatment strategies, and the establishment of an effective animal model is the basis of the study of BPD. Therefore, this study proposes that the animal model of BPD in premature rats can be established successfully when they are exposed to high concentration of oxygen, in order to provide a new theoretical model for further study on the prevention and treatment of BPD.

## Materials and Methods

### Ethics Approval

The use and care of laboratory rodents was performed according to the Animal Laboratory Center of Pediatrics, Children's Hospital of Fudan University and approved by the Committee of Animal Laboratory Management and Ethics, Shanghai Children's Hospital.

### Experimental Animals

250–300 g healthy adult Specific pathogen Free (SPF) grade Sprague-Dawley (SD) rats, 25 females and five males, provided by Shanghai Sipur-Bikai experimental animal Co., Ltd., animal license No. SCXK (Shanghai) 2018-0006.

### Cage Closing and Conception of Rats

Male and female SD rats were fed in metabolic cages at 1:5. The mat and excrement of the cage were checked at 6 a.m. every day. Check the vaginal plug of females, and the day that vaginal plug was checked as the day 0 of pregnancy.

### Cesarean Section and Milk Substitute of Premature Rats

On the 21st day of gestation, pregnant SD rats were subjected to cesarean sections. The anesthetics (pentobarbital sodium 10 mg diluted in normal saline 20 ml) were intramuscularly injected at the dose of 30–50 mg/kg. The induced anesthetics were given at 30 mg/kg.

**Figure 1:**
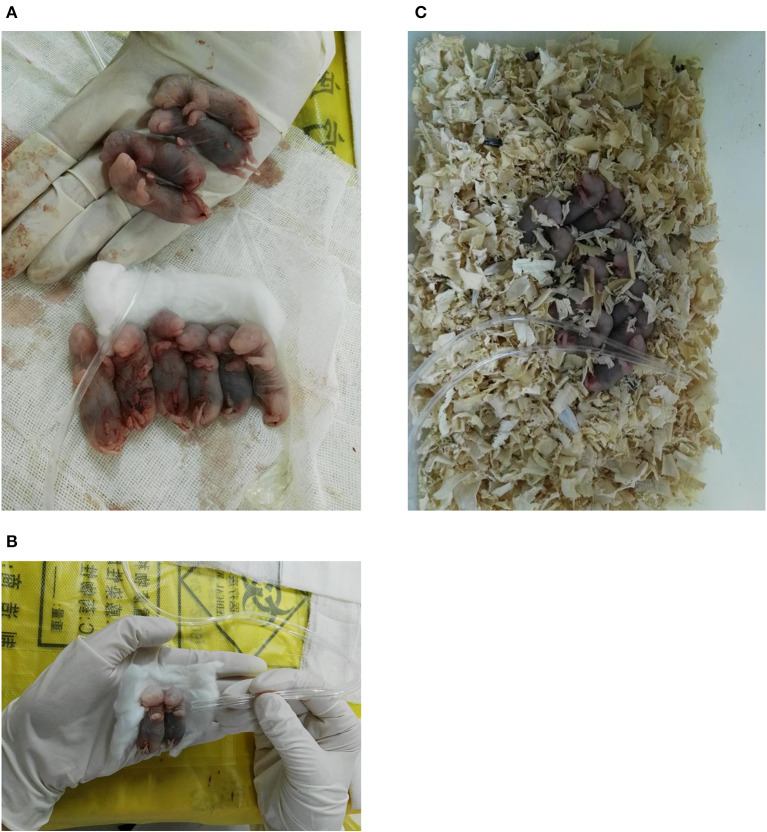
**(A-C)** The premature rats were placed in forage and resuscitated with 100% oxygen by operator.

The abdomen was sectioned, and the uterus were pulled out gently after anesthesia. The operator pulled out the uterus gently, and took out the fetal rats quickly. Continue to press the chest of premature rats (60–100 times/min) and give 100% oxygen for 30 min by the operator. The premature rats were fed with the breast milk by the mother rats which delivered naturally (Figures 1A-C).

### Premature Rats Exposed to Hyperoxia

Eighty preterm rats were randomly divided into two groups 24 h after birth: the air group and the hyperoxia group. The air group was exposed to room air, and the hyperoxia group was exposed to oxygen concentration with 80 ± 5%. At 1, 4, 7, 10, and 14 days after hyperoxia exposure, the lungs of preterm rats were embedded in paraffin and made into 5 μm sections for HE staining.

### General and Pathological Observation of Lung Tissue

All analyses were performed by the same author. The color of the lung surface was observed by naked eyes, and the morphology of the histology slides were analyzed by Hematoxylin-Eosin (HE) staining.

### Morphology of Lung Tissue

Morphological changes of lung tissue structure at different time points: the basic morphology of lung tissue, alveolar septal thickness, degree of alveolalization, inflammatory cell infiltration were observed under microscope.

Radial alveolar count (RAC): a vertical line was drawn from the center of respiratory bronchioles to the nearest pleura or fibrous septum. The number of alveoli on this line is called the RAC. One section was randomly selected from each preterm rat and observed under microscope (X 100). Five visual fields were randomly selected from each section to calculate the average number, reflecting the number of alveoli in the end respiratory unit.

### Statistical Analysis

The experimental data were analyzed by spss20.0 statistical software. The data were expressed by x ± s. The results of each time point in the two groups were tested by oneway ANOVO, and the results of air group and hyperoxia group were tested by two independent samples *T*-test. *p* < 0.05 was statistically significant.

## Result

### General Condition of Premature SD Rats

Fifteen SD female rats were successfully pregnant, and 116 premature rats were produced during the operation. Twelve premature rats died due to various reasons such as hemorrhage, poor resuscitation, and rescue effect, feeding difficulties in 24 h after the operation, trampled by adult rats, and so on. Finally, 80 premature rats were divided into two groups: the air group and the hyperoxia group. There was no death in the air group, three premature rats died in the hyperoxia group on the 4th day. Each group was divided into five subgroups, the 1, 4, 7, 10, and 14-days. Each subunit contained eight rats.

**Figure 2:**
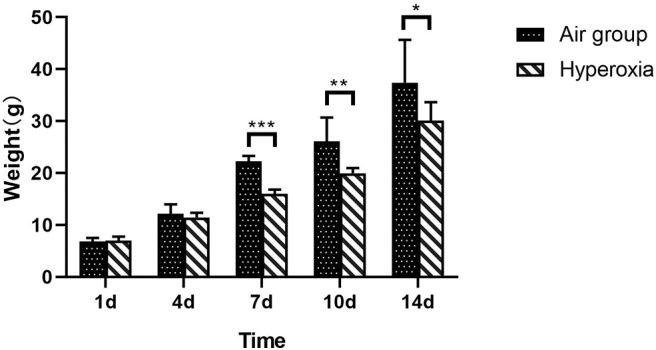
Comparison of weight between two groups at different time. Compare with the air group, weight of premature rats in hyperoxia group at 1d, and the weight of the rats in the hyperoxia group increased slowly at the 7, 10, and 14d, which was statistically significant (^*^p < 0.05, ^**^p < 0.01, ^***^p < 0.001).

**Figure 3:**
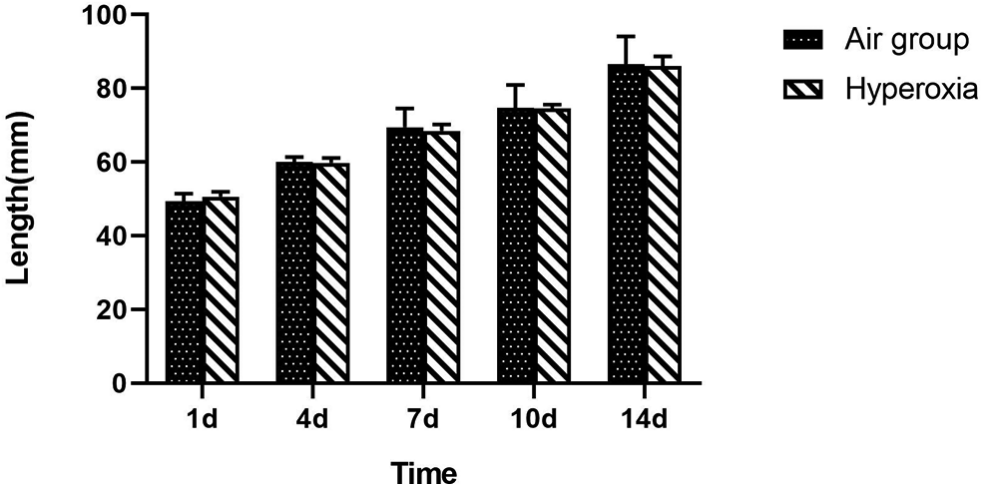
Comparison of length between two groups at different time. Compare with the air group, there was no difference in length of the premature rats in the hyperoxia group, shows that the premature rats exposed to hyperoxia did not affect the development of length.

There was no significant difference in weight between air group and hyperoxia group at 1d (*p* = 0.679). In the air group, the rats had good response, rapid weight growth, and normal growth and development. In the hyperoxia group, the rats began to show poor response, decreased autonomous activity, increased respiratory rate, cyanosis of mouth, nose, and limbs, and other dyspnea symptoms gradually increased in the 4d. At 10d, the rats in the hyperoxia group appeared whole body twitch and head tremor without oxygen, and recovered to normal after oxygen supplied. Compared with the air group, the weight of the rats in the hyperoxia group increased slowly at 7, 10, 14 days, the difference was statistically significant (*p* < 0.05) (Table 1 and Figure 2). At the same time, the color of hair was poor and the time of opening eyes was prolonged. There was no significant difference between the air group and the hyperoxia group in length (*p* > 0.05) (Table 2 and Figure 3).

**Table 1:** Comparison of weight between two groups at different time (*g*).

**Groups**	***n***	**1d**	**4d**	**7d**	**10d**	**14d**	***F***	***p***
Air group	8	6.85 ± 0.65	12.18 ± 1.80	22.27 ± 1.05	26.09 ± 4.60	37.40 ± 8.25	99.914	<0.001
Hyperoxia	8	7.00 ± 0.79	11.39 ± 0.96	16.00 ± 0.83	19.96 ± 1.03	30.12 ± 3.53	338.388	<0.001
*t*		−0.422	1.909	15.247	3.894	2.335		
*p*		0.679	0.063	<0.001	0.004	0.044		

**Table 2:** Comparison of length between two groups at different time (mm).

**Groups**	***n***	**1d**	**4d**	**7d**	**10d**	**14d**	***F***	***p***
Air group	8	49.40 ± 2.07	60.00 ± 1.41	69.47 ± 5.08	74.67 ± 6.30	86.62 ± 7.53	84.363	<0.001
Hyperoxia	8	50.60 ± 1.35	59.77 ± 1.36	68.47 ± 1.77	74.53 ± 1.06	86.00 ± 2.71	684.800	<0.001
*t*		−1.538	0.415	0.721	0.081	0.245		
*p*		0.154	0.682	0.481	0.936	0.809		

### Histopathology of Lung Tissue in Two Groups of Preterm Rats

**Figure 4:**
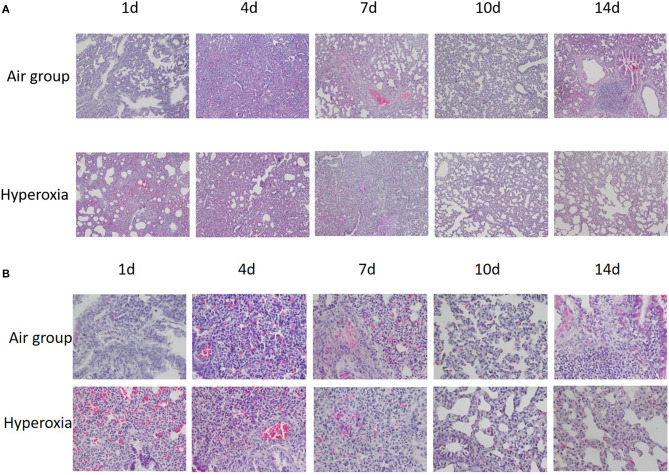
**(A)** HE staining of lung tissue at different time in two groups (×100). **(B)** HE staining of lung tissue at different time in two groups (×400). When HE stained lung tissue was placed under 100 and 400 times light microscope, it can be seen that compared with the air group, the alveoli in the hyperoxia group lost their normal regular shape and structure disorder, the alveoli space became thinner, the alveoli cavity increased, and the number of alveoli decreased, especially at 10 and 14d.

Generally speaking, the lung tissue of the two groups was mainly composed of pulmonary interstitium at 1d, the respiratory and circulatory vascular system was immature, the respiratory bronchioles, and their alveoli were less, the lung parenchyma gradually increased and the interstitial decreased at the 4d, the number of alveoli gradually increased, and the pulmonary bronchi and vascular system at all levels were refined and increased. In the air group, the structure and morphology of alveoli were clear and regular, the alveoli were well-developed, the size of alveoli was uniform, the walls of alveoli were smooth, the alveoli space was thin, no inflammation or exudate was found in the alveoli. Under the light microscope, the alveoli in the hyperoxia group lost normal regular shape and disordered structure, the alveoli became thinner and enlarged, the number of alveoli decreased, especially at 10 and 14d (Figure 4).

### RAC of Lung Tissue in Two Groups of Preterm Rats

**Figure 5:**
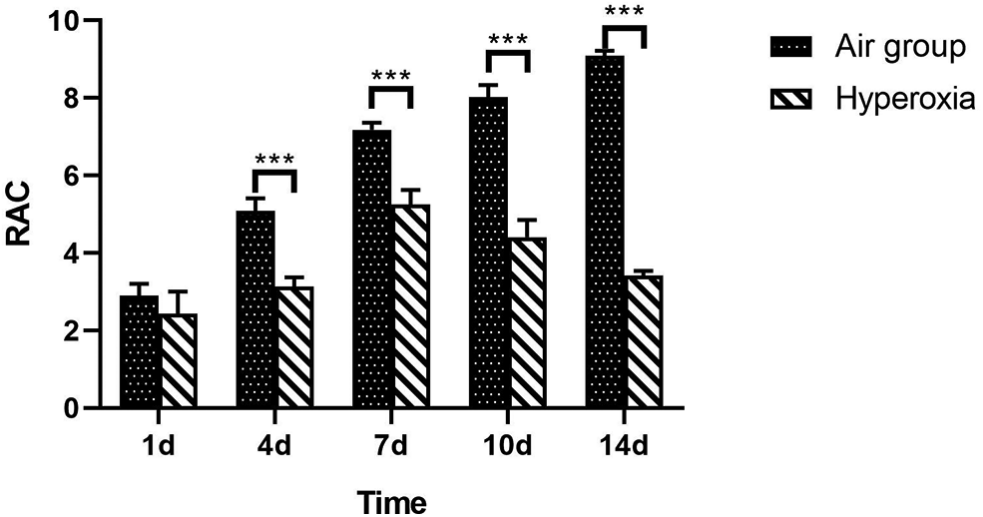
RAC of lung tissue in two groups at different time points. From the center of respiratory bronchioles to the nearest pleura or fibrous septum, a vertical line is drawn. The number of alveoli on this line is the RAC, which reflects the development of alveoli. Compare with the air group, the RAC of hyperoxia group was significantly lower than that of the air group (*p* < 0.05). The RAC of the air group increased while the hyperoxia group decreased gradually after 7 days. ^***^p < 0.001.

At each time point, the RAC of hyperoxia group was significantly lower than that of air group (*p* < 0.05), and the RAC of air group gradually increase, while the hyperoxia group gradually decreased (Table 3 and Figure 5).

**Table 3:** RAC of lung tissue in two groups at different time points.

**Groups**	***n***	**1d**	**4d**	**7d**	**10d**	**14d**	***F***	***p***
Air group	8	2.90 ± 0.30	5.09 ± 0.32	7.18 ± 0.18	8.03 ± 0.31	9.09 ± 0.13	720.663	<0.001
Hyperoxia	8	2.44 ± 0.56	3.14 ± 0.23	5.25 ± 0.38	4.41 ± 0.44	3.41 ± 0.13	63.987	<0.001
*t*		2.083	11.989	13.069	18.961	87.910		
*p*		0.056	<0.001	<0.001	<0.001	<0.001		

## Discussion

In recent years, with the improvement of treatment for premature infants, the survival rate of premature infants, especially very low birth weight infants (VLBWI) and extremely low birth weight infants (ELBWI) has increased significantly (11), and the incidence rate of bronchopulmonary dysplasia has increased (12). BPD is a kind of chronic lung disease (CLD) that causes the poor long-term prognosis in premature infants. Because the children with BPD need longer hospitalization and long-term oxygen therapy, it has many adverse effects on family and society, it is important to research the etiology, pathophysiology, and the corresponding prevention and treatment measures of BPD. To establish an effective and reliable animal model of BPD is the first problem to be solved by researchers. The main inducing factors of BPD animal model are hyperoxia, mechanical ventilation injury, intrauterine inflammation, postnatal continuous hypoxia, and intrauterine hypoxia (13). At present, it is considered that oxidative stress is one of the main risk factors for BPD, and its pathogenesis is the interruption of lung development through the mechanism of interrupting growth factor signal transduction, cell proliferation, apoptosis, and angiogenesis (14). In 2014, Yang et al. observed that the development of alveoli in lung tissue was weakened, and isolated alveolar epithelial cells (AT2 cells) showed epithelial mesenchymal transition (EMT) in newborn rats by hyperoxia exposed for 21 h after birth (15). Therefore, up to now, the animal model of BPD induced by hyperoxia is still the most commonly used.

The development of lung in human usually goes through five stages, including embryonic stage, pseudoglandular stage, tubule stage, vesicle stage, and alveolar stage (16). The relevant time point of lung development is located in the vesicular phase, usually between the 24 and 38th weeks of pregnancy (corresponding to d0, d4) (17). In the alveolar type II epithelial cells of the full-term rats, the existence of pulmonary surfactant indicates that the lung tissue of the full-term rats is functionally mature (18). The development of lung in premature rats (gestational age 21 days) is in the initial stage of the vesicle, which is closer to the stage of BPD in premature infants. At present, BPD animal models are mainly come from hyperoxia induced animal models, which rodents are the most widely used. As early as 1932, some researchers place newborn rats in an environment having an 83.6% oxygen concentration. A month later, there are thickening and hyalinization of the walls with ultimate thrombosis of many in the lung tissue of rats (19). In the next 100 years, many studies established the animal model of BPD by exposing newborn rats to high concentration of oxygen. In 1978, Yam et al. detected the increase of superoxide dismutase (SOD), glutathione peroxidase (GP), glutathione reductase (GR), and glutathione (GSH) in newborn rats increased by exposing to hyperoxia, which proved that the above substances increased the tolerance of lung tissue to hyperoxia injury, and successfully established a BPD animal model of newborn rats with hyperoxia (20). Recent studies have shown that newborn rats are exposed to different concentrations of oxygen (60, 80, and 100%, respectively), and the probability of BPD is not the same. When FiO_2_ is <60%, the probability of BPD is low (21). In this study, the premature SD rats were used as the experimental objects to make the animal model of BPD by exposing to hyperoxia, which has the advantages of easy access to rodents, relatively short pregnancy cycle and convenient management. At present, most of the known experimental animal models of BPD are full-term animal models, and a few are preterm animal models (22). The smaller the gestational age, the higher the severity of BPD, suggesting that premature is another important risk factor for BPD (23). The development of lung in the 21-days pregnant SD rats was in the period of vesicle during delivery, similar to that of the premature infants (24). A number of studies at home and abroad have proved that hyperoxia exposure causes the structural abnormality of pulmonary microvasculature in rats, which is similar to the pathological changes of lung tissue in premature infants with BPD (25). In 1998, the premature rats delivered by cesarean section on the 21st day of pregnancy were exposed to high concentration of oxygen. By comparing the production of pulmonary surfactant, it was confirmed that premature exposure to more than 95% of oxygen for 7–14 days, the clinical morphological characteristics of lung in preterm rats was similar to that of human premature infants (26). Similarly, Zhu et al. proposed that hyperoxia induce lung injury in preterm rats, in order to explore the dynamic expression and role of SUMO modified C/EBP α in BPD (27). In this experiment, premature SD rats were used as the experimental objects. The rats in hyperoxia group were exposed to high concentration oxygen at 0, 4, 7, 10, 14d, respectively. The experimental results showed that the animal model was established successfully by comparing the weight, length, pathological changes of lung tissue, and RAC count of premature rats, the results have continuity in time as well. Postnatal growth restriction (PTGR) and hyperoxia can reduce the VEGF signal conduction in the lung, and cause abnormal pulmonary vascular and alveolar development in premature and rodent models, resulting in bronchopulmonary dysplasia, and pulmonary hypertension (28). Some studies have confirmed that malnutrition in the early postnatal period will increase the risk of BPD in premature infants (29), so the impact of PTGR on the lungs cannot be ignored.

In this study, compare with the air group, the premature rats in hyperoxia group showed poor response, decreased activity, and dyspnea with the increase of exposure to hyperoxia, and the weight growth was slow from the 7d, the difference was statistically significant (*p* < 0.05), However, there was no significant difference between the two groups in length (*p* > 0.05). Histopathology of lung tissue showed that the alveoli in hyperoxia group lost normal regular shape and structure disorder, the alveoli became thinner and the alveoli cavity enlarged, the number of alveoli decreased, especially on the 10d and 14td, and the RAC was significantly lower than that in air group (*p* < 0.05), these changes are consistent with the pathological changes of BPD in preterm infants (30), indicating that we has successfully established an animal model of hyperoxia exposure to BPD.

The animal model of BPD provides an important basis for the study of the possible mechanism and prevention and control strategy of the disease. However, due to the diversity of the etiology of BPD, there are many ways to establish the animal model of BPD. It is still necessary to further study to find a simpler and more similar with premature infants in BPD. But no matter which animal model is used, it needs to combine the clinical significance to make the experimental data better to reduce the disease.

## Data Availability Statement

The data sets used and/or analyzed during the current study are available from the corresponding author on reasonable request.

## Ethics Statement

The animal study was reviewed and approved by Committee of Animal Laboratory Management and Ethics, Shanghai Children's Hospital.

## Author Contributions

XZ, CC, and XG made substantial contributions to the conception and design, acquisition of data, or analysis, and interpretation of data. XZ and CC were involved in drafting the manuscript or revising it critically for important intellectual content. CC, XG, BW, and XC revised the manuscript and gave final approval of the version to be published. The authors agree to be accountable of the version to be published. The authors agree to be accountable for all aspects of the work in ensuring that questions related to the accuracy or integrity of any part of the work are appropriately investigated and resolved. All authors read and approved the final manuscript.

## Conflict of Interest

The authors declare that the research was conducted in the absence of any commercial or financial relationships that could be construed as a potential conflict of interest.
